# Traction clip closure of small bowel perforation during single-balloon enteroscopy in a patient with surgical altered anatomy

**DOI:** 10.1055/a-2663-8490

**Published:** 2025-08-14

**Authors:** Koichi Soga, Suguru Miyo, Fuki Hayakawa, Mayumi Yamaguchi, Masaru Kuwada, Ikuhiro Kobori, Masaya Tamano

**Affiliations:** 1 26263 Department of Gastroenterology, Dokkyo Medical University Saitama Medical Center, Koshigaya, Japan

Endoscopic retrograde cholangiopancreatography (ERCP) of surgically altered gastrointestinal anatomy (SAA) remains technically challenging. ERCP perforation rates in SAA are 2–5% 1 . Endoscopists should familiarize themselves with troubleshooting techniques to effectively manage these complications.

A 69-year-old male with a history of distal gastrectomy followed by Roux-en-Y reconstruction presented 2 months postoperatively with acute pancreatitis owing to common bile duct stones (CBDs). Pancreatitis resolved conservatively, and elective management of the residual CBDs was planned.

ERCP was performed using a single-balloon enteroscope (SIF-H290S; Olympus, Tokyo, Japan). Multiple CBDs were visualized and extracted using cholangiography and an endoscopic mechanical lithotripsy device, respectively ( Fig. 1 ).

**Fig. 1:**
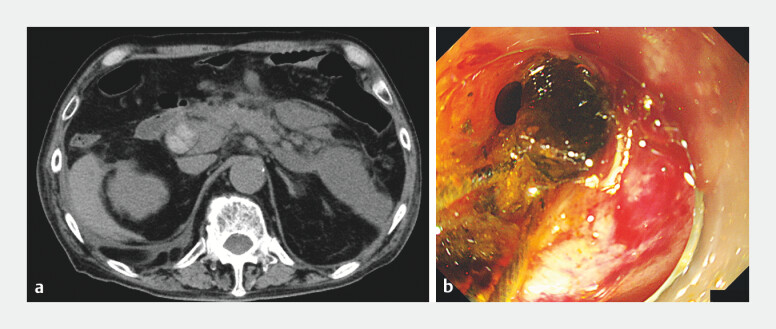
Computed tomography (CT) and endoscopic management of common bile duct (CBD) stones. Procedural sequence of CBD stone management during single-balloon enteroscopy in the present case. **a** Abdominal CT scan showing multiple stones in the CBD. **b** Endoscopic mechanical lithotripsy performed to fragment the CBD stones.

However, while attempting to extract residual stones using a balloon catheter, inappropriate endoscopic manipulation caused extensive mucosal lacerations and perforations. The mucosal laceration and subsequent perforation were attributed to excessive traction and rotational manipulation of the endoscope during balloon catheter-assisted stone extraction. Although such maneuvers are commonly performed during endoscopic removal of CBDs with a side-viewing duodenoscope, they are not necessarily required in procedures involving SAA.

Owing to the fragile and ischemic nature of the injured mucosa, initial closure attempts using clips were inadequate. A traction clip technique using silicone bands (SureClip Traction Band; Micro-Tech, China) was employed because of the high risk of complete closure failure and/or delayed perforation expansion 2 . Traction was initially established between two healthy mucosal sites away from the perforation using traction clips to create a stable base. Additional clips were sequentially placed, enabling robust closure of the defect without exacerbating tissue injury ( Fig. 2 , Fig. 3 , Fig. 4 , Video 1 ). Complete suturing was safely performed. Clinical management with conservative therapy resulted in a successful recovery without further complications.

**Fig. 2:**
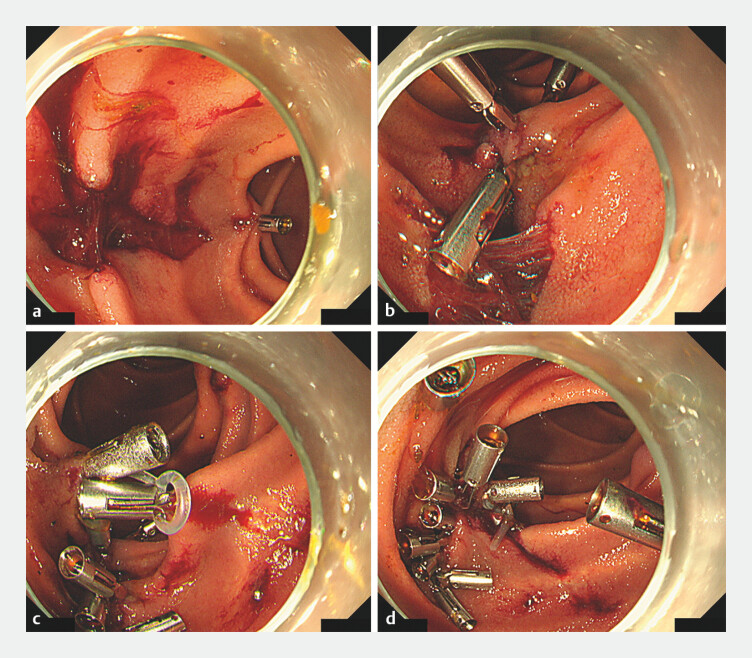
Endoscopic closure of mucosal laceration using the traction clip technique. Endoscopic management of small intestinal mucosal laceration and perforation induced during CBD stone extraction using the traction clip technique with silicone bands. **a** Longitudinal mucosal laceration observed during the procedure. **b** Initial closure attempts with clips were inadequate owing to the fragile and ischemic nature of the injured mucosa. **c** A traction clip technique utilizing silicone bands (SureClip Traction Band, Micro-Tech, China) was employed owing to the high risk of delayed perforation expansion. Traction was first established between two healthy mucosal sites away from the perforation, providing a stable base. **d** Additional clips are placed in sequence, enabling robust closure of the defect without exacerbating tissue injury.

**Fig. 3:**
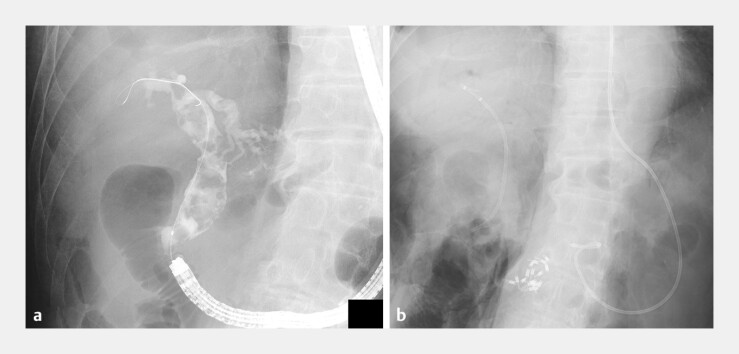
Fluoroscopic confirmation of stone removal and clip placement. Fluoroscopic images showing multiple CBD stone extractions and clip placements after closure of the perforation site. An endoscopic nasobiliary drainage (ENBD) tube (Flexima, 7.5 Fr; Boston Scientific, USA) is placed to drain the perforated area.

**Fig. 4:**
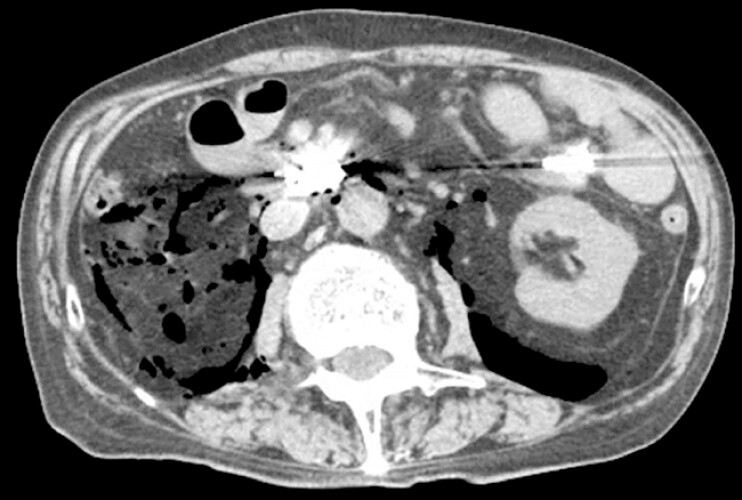
Postprocedural CT findings of mucosal closure and retroperitoneal free air. Postprocedural abdominal CT scan is performed. The CT image shows endoscopic clips securing the mucosal defect and free air in the retroperitoneal space.

Traction clip closure of small bowel perforation during single-balloon enteroscopy in a patient with surgical altered anatomy.Video 1

Previous studies have described various endoscopic techniques using traction clips for gastrointestinal defect closure, particularly after endoscopic submucosal dissections 2 3 . The traction clip technique reduces tension at the closure site, facilitates healthy tissue approximation, and enhances repair durability. This method may reduce the risk of delayed perforation, particularly in thin-walled structures, including the small intestine. Our experience suggests that this technique is particularly beneficial for managing ERCP-related small-bowel perforations in patients with SAA.

Endoscopy_UCTN_Code_CPL_1AI_2AD

## Notes

Endoscopy E-Videos https://eref.thieme.de/e-videos
*E-Videos* is an open access online section of the journal *Endoscopy* , reporting on interesting cases and new techniques in gastroenterological endoscopy. All papers include a high-quality video and are published with a Creative Commons CC-BY license. Endoscopy E-Videos qualify for HINARI discounts and waivers and eligibility is automatically checked during the submission process. We grant 100% waivers to articles whose corresponding authors are based in Group A countries and 50% waivers to those who are based in Group B countries as classified by Research4Life (see: https://www.research4life.org/access/eligibility/ ). This section has its own submission website at https://mc.manuscriptcentral.com/e-videos .
